# Editorial: Psychoactive natural products: Potential in the treatment of brain diseases and strategies to improve delivery

**DOI:** 10.3389/fphar.2022.1032496

**Published:** 2022-10-03

**Authors:** Célia Cabral, Claudia Carbone, Pamela Maher, Miguel Castelo-Branco

**Affiliations:** ^1^ Faculty of Medicine, Clinic Academic Center of Coimbra (CACC), University of Coimbra, Coimbra Institute for Clinical and Biomedical Research (iCBR), Coimbra, Portugal; ^2^ Center for Innovative Biomedicine and Biotechnology (CIBB), University of Coimbra, Coimbra, Portugal; ^3^ Department of Life Sciences, Center for Functional Ecology, University of Coimbra, Coimbra, Portugal; ^4^ Department of Drug and Health Sciences, University of Catania, Catania, Italy; ^5^ Salk Institute for Biological Studies, La Jolla, IL, United States; ^6^ Coimbra Institute for Biomedical Imaging and Translational Research (CIBIT), Coimbra, Portugal

**Keywords:** psychoactive natural products, brain diseases, new molecules, new therapies, drug delivery

For this special issue, focusing on psychoactive natural products with therapeutic potential for the treatment of brain diseases, we have invited authors to contribute with research and review articles illustrating and stimulating the continuing effort in drug discovery and drug development using natural products as a source of bioactive molecules.

From submissions, four high-quality manuscripts (3 research papers and 1 systematic review/metanalysis) were selected covering the above-mentioned topics. These articles covered both animal and human research with quite distinct methodological approaches. Interestingly some common themes emerge from these works.

Kiraga et al. studied the persisting effects of Ayahuasca on empathy, creative thinking, decentering, personality, and well-Being. They evaluated the effects in volunteers attending ayahuasca ceremonies that were asked to complete a test battery at three separate occasions: baseline, the morning after, and 1 week after the ceremony. In total, 43 attendees (males = 22; females = 21) completed parts of the baseline assessment, 20 (males = 12; females = 8) completed assessments in the morning after the ceremony, and 19 (males = 14; females = 5) completed assessments at the 1-week follow-up. The study suggests that a single ingestion of ayahuasca in a social setting is associated with an enhancement in subjective well-being, an increased ability to take an objective and non-judgmental stance towards the self (decentering), and the ability to correctly recognize emotions in others, compared to baseline, lasting up to 1-week post-ceremony. To understand the therapeutic potential related to these effects, further research with clinical populations is needed, including its link with therapeutic outcomes.

Henriques et al. investigated the effects of repeated treatment with ibogaine on the reinstatement of conditioned place preference (CPP) to ethanol in male mice. Both the rewarding effects of ethanol (1.8 g/kg, i. p.) or ibogaine (10 or 30 mg/kg, p. o.) were investigated in the CPP model as well as the effects of repeated treatment with ibogaine (10 or 30 mg/kg, p. o.) on the reinstatement of ethanol-induced CPP. Their results showed that ethanol, but not ibogaine, induced CPP in mice. Treatment with ibogaine after conditioning with ethanol blocked the reinstatement of ethanol-induced CPP, both during a drug priming reinstatement test and during a drug-free test conducted after re-exposure to ethanol in the ethanol-paired compartment. These findings add to the literature suggesting that psychedelics, in particular ibogaine, may have therapeutic properties for the treatment of alcohol use disorder at doses that do not have rewarding effects *per se*.

Castelhano et al. performed a systematic review on the effects of tryptamine psychedelics in the brain. They investigated the question of whether changes in activation patterns and connectivity map onto regions with larger 5HT1A/5HT2A receptor binding, as expected from indoleamine hallucinogens (in spite of the often reported emphasis only on 5HT2AR). They found that regions with changed connectivity and/or activation patterns matched regions with a high density of 5HT2A receptors, namely visual BA19, visual fusiform regions in BA37, dorsal anterior and posterior cingulate cortex, medial prefrontal cortex, and regions involved in theory of mind such as the surpramarginal gyrus, and temporal cortex (rich in 5HT1A receptors). However, they also found relevant patterns in other brain regions such as dorsolateral prefrontal cortex. Moreover, many of the above-mentioned regions also have a significant density of both 5HT1A/5HT2A receptors, and available PET studies on the effects of psychedelics on receptor occupancy are still quite scarce, precluding a metanalytic approach. Finally, they found a robust neuromodulatory effect in the right amygdala. In sum, the available evidence points towards strong neuromodulatory effects of tryptamine psychedelics in key brain regions involved in mental imagery, theory of mind and affective regulation, pointing to potential therapeutic applications of this class of substances.

Tan et al. investigated the effects of therapy with Shugan Jieyu capsules (SG) on the function of gut microbiota and Intestinal microbiota in rats with chronic unpredictable mild stress (CUMS)-induced depression. With treatment of SG, the depression-like behaviors of CUMS-induced rats were reversed; the corticosterone levels and the adrenal index decreased significantly; the level of serotonin increased significantly; and the alpha and beta diversity analysis of microbiota showed an increase in the richness and uniformity of the flora. SG regulated the relative abundance of Actinobacteria, Erysipelotrichaceae, Bifidobacteriaceae, Atopobiaceae, Dubosiella, and Bifidobacterium. Linear discriminant analysis effect size analysis demonstrated that Lactobacillaceae (family level), *Lactobacillus* (genus level), Lactobacillales (order level), Bacilli (class level), and Lactobacillus-reuteri (species level) were biomarkers in the SG group samples, and also likely to modulate metabolic pathways, such as those involved in carbohydrate metabolism, amino acid metabolism, and signal transduction. These data clearly illustrated the effect of SG in gut microbiome, thus laying the foundation for uncovering more insights on the therapeutic function of traditional Chinese antidepressants. The potential of SG on mechanisms of antidepression to alter gut microbiota and intestinal microbiome function exposed to CUMS should be explored in the future.

As common themes, both human studies (Castelhano et al.; Kiraga et al.) address the effects of tryptamine psychedelics, from the behavioral and neural response points of view. Interestingly, they converge on the role of these compounds in theory of mind and affective regulation processes for complementary points of view. The role of central serotonin receptors seems to be pivotal in these effects. Interestingly, peripheral changes in serotonin seems to be associated with the soothing effects of Shugan Jieyu capsules. The study using ibogaine focuses on a potential role on prevention of alcohol addiction even without substantial effects on the reward system. This finding is consistent with the observations of Castelhano et al. suggesting a dissociation between limbic and reward system responses. Interestingly, the prominent reduction of amygdala activity suggests a therapeutic target for anxiety or stress reduction using these compounds.

Future directions of research should address how these compounds can modulate brain plasticity and if a small number of sessions is sufficient to induce such plasticity. Appropriate combination of functional and molecular imaging methods might be necessary to probe such effects *in vivo* (Rebelo et al., 2021).

In sum these exciting studies pave the way for the development of novel therapeutic approaches based on psychoactive natural products.
